# Blue light stimulated-autofluorescence green flash of lysosome-related organelles in the intestinal cells of nematodes

**DOI:** 10.1093/g3journal/jkaf209

**Published:** 2025-09-04

**Authors:** Chieh-Hsiang Tan, Keke Ding, Mark G Zhang, Paul W Sternberg

**Affiliations:** Division of Biology and Biological Engineering, California Institute of Technology, Pasadena, CA 91125, United States; Division of Biology and Biological Engineering, California Institute of Technology, Pasadena, CA 91125, United States; Division of Biology and Biological Engineering, California Institute of Technology, Pasadena, CA 91125, United States; Division of Biology and Biological Engineering, California Institute of Technology, Pasadena, CA 91125, United States

**Keywords:** lysosome-related organelles, gut granules, autofluorescence, light stimulation, *Caenorhabditis elegans*, *Steinernema hermaphroditum*, *Oscheius tipulae*, *Panagrellus redivivus*, *Pristionchus pacificus*, *Panagrolaimus*

## Abstract

The lysosome-related organelles (“gut granules”) in the intestinal cells of many nematodes, including *Caenorhabditis elegan*s, play an important role in metabolic and signaling processes, but they have not been fully characterized. We report here a previously undescribed phenomenon in which the autofluorescence of these granules displays a “flash” phenomenon in which fluorescence decreases are preceded by sharp increases in fluorescence intensity that expand into the surrounding area when the granules are stimulated with blue light. Autofluorescent granules are present in the intestinal cells of all 6 nematode species examined, with differences in morphology and distribution pattern. Five species exhibit the flash phenomenon: *Panagrellus redivivus* (Clade IV), *Steinernema hermaphroditum* (Clade IV), *C. elegans* (Clade V), *Oscheius tipulae* (Clade V), and *Pristionchus pacificus* (Clade V). The reaction of the granules to blue light stimulation greatly differs among different developmental stages and may also be dependent on physiological conditions. In addition, even within the same animal, the sensitivity of individual granules differs, with some of the variation associated with other characteristics of the granules, such as their overall location within the intestine. We hypothesize that the differences in response to blue light indicate the existence of different subpopulations of gut granules in nematode intestines, and the visually spectacular dynamic fluorescence phenomenon we describe might provide a handle on their eventual characterization.

## Introduction

The intestinal cells of *Caenorhabditis elegans* (Maupas 1900) contain a type of organelle known as gut granules, suggested to be the rhabditin granules observed in many other nematodes (Maupas 1900; Cobb 1914; Hedrick 1935; Chitwood and Chitwood 1950; Thomas and Quastler 1950; Laufer et al. 1980). The birefringent and autofluorescent gut granules are robustly present in intestinal cells and intestinal precursor cells (Laufer et al. 1980; Hermann et al. 2005) and are so prominent that they have served as useful markers for the intestinal cell lineage in *C. elegans* development. Based on morphological, biochemical, and genetic evidence, these granules are considered lysosome-related organelles (LROs; Clokey and Jacobson 1986; Kostich et al. 2000; Hermann et al. 2005; Delevoye et al. 2019). While far from being fully characterized, previous studies found these organelles to be associated with metabolism and homeostasis processes such as the storage of fat, cholesterol, and heme (Schroeder et al. 2007; Lee et al. 2015; Sun et al. 2022) and the storage and sequestering of trace metals (Roh et al. 2012; Chun et al. 2017). Gut granules are also known to be involved in the biogenesis of small signaling molecules, such as ascarosides, and in the resistance to stress and pathogens (Le et al. 2020; Hajdu et al. 2023).

In this study, we found that some autofluorescent granules in nematode intestines underwent a rapid and dynamic change in fluorescence intensity when observed in the GFP channel (blue light excitation; green emission). In *C. elegans*, this phenomenon depends on the worm's ortholog of human Rab32/38, *glo–1* (*G*ut granule *LO*ss) (Hermann et al. 2005; Morris et al. 2018), which has been shown to be required for gut granule biogenesis, suggesting that gut granules were the source of the autofluorescent green flash. High-resolution confocal imaging of ex vivo, partially exposed *C. elegans* intestines revealed the spatial-temporal patterns of the fluorescence intensity changes, which we found to be different under the green and red channels. We reasoned that this suggests the existence of at least 2 distinct types of fluorophores or fluorophore transitions in the gut granules. Green fluorescence intensities in the granules sharply increased, rapidly diffused, and then dissipated, a phenomenon that we describe as “gut granule green flash.” No similar rapid increase in red fluorescence intensities was observed prior to their simultaneous dissipation. By increasing the intensity of the (GFP) excitation wavelength blue light during imaging, we were able to significantly reduce the amount of time between the start of the light exposure and the onset of gut granule green flash, suggesting that the phenomenon is likely induced by blue light. We found that their responses to blue light stimulations differ greatly among different developmental stages, in which we were able to reliably document the phenomenon in the second (L2), fourth (L4), and the stress-resistant dauer larvae stages, but were unable to do so in the L1 stage and adult animals. The association of the phenomenon with the availability of food in ex vivo settings suggests that it may also be influenced by physiological conditions. To further understand the green flash phenomenon, we investigate the autofluorescent granules in 5 other nematode species. Including 2 other clade V nematodes (based on the 5 major clade classification of Blaxter et al. (1998)), namely *Oscheius tipulae* (Lam and Webster 1971) and *Pristionchus pacificus* (Sommer et al. 1996), as well as 3 clade IV worms, namely *Steinernema hermaphroditum* (Stock et al. 2004), *Panagrellus redivivus* (Linnaeus), and a species of the *Panagrolaimus* genus (*Panagrolaimus* sp. PS1159) (Sommer and Sternberg 1996). We found autofluorescent granules to be present in the intestinal cells of all 5 species, 4 of which exhibit the green flash phenomenon with different patterns. From these observations, we conclude that it is likely that, at least in some developmental stages, gut granules comprise distinct subgroups, literally highlighted by fluorescence dynamics.

## Materials and methods

### Nematode strain maintenance and general methods

The culture and maintenance of *C. elegans*, *O. tipulae*, *P. pacificus*, *P. redivivus*, and *Panagrolaimus* sp. PS1159 was done similarly to the standard *C. elegans* procedures as described in Brenner (1974). Briefly, worms were cultured on 6 cm Petri dishes containing Nematode Growth Medium (NGM) agar with a bacterial lawn of *Escherichia coli* strain OP50. *C. elegans*, *O. tipulae*, *P. pacificus*, and *P. redivivus* were maintained at 20 °C, and *Panagrolaimus* sp. PS1159 at 25 °C. The culture and maintenance of *S. hermaphroditum* were done similarly to the procedures described in Cao et al. (2022) and Rodak et al. (2024). Briefly, worms were either cultured on 10 cm Petri dishes containing NGM agar with a bacterial lawn of *Xenorhabdus griffiniae* strain HGB2511 or 10 cm Petri dishes containing Enriched Peptone Medium (EPM) with a bacterial lawn of *Comamonas aquatica*  DA1877 (Avery and Shtonda 2003) at 25 °C. For *C. elegans*, the Bristol N2 strain (Brenner 1974) was used as the wild-type reference strain and is the strain from which the *glo–1* mutant strain was derived. The 5 times out-crossed *glo-1(zu391*lf*)* X (Hermann et al. 2005) mutant strain OJ1347 (Wang et al. 2013) was a gift from Dr. Derek Sieburth of the University of Southern California. For *O. tipulae*, *P. pacificus*, *S. hermaphroditum*, *P. redivivus*, and *Panagrolaimus* sp., the following wild-type reference strains were used, respectively: CEW1 (Winter 1992), PS312 (Sommer et al. 1996), PS9179 (Cao et al. 2022), MT8872 (Sternberg and Horvitz 1981), and PS1159 (Sommer and Sternberg 1996).

### Preparation of *C. elegans* dauer larvae

To induce dauers, 10 to 20 young adults were placed on 35 mm diameter Petri dishes containing 2 mL of NGM agar (without peptone) supplemented with a quantity of crude pheromone extract (Schroeder and Flatt 2014) that normally induced 95% to 100% of dauers in wild-type animals—typically 10 to 30 µL per 2 mL of agar. Plates were seeded with 10 µL of 8% w/v *E. coli*  OP50 that had been heat-killed at 95 °C for 5 min. Adults were picked onto the plate and allowed to lay eggs at room temperature (RT; 22 to 23 °C) for 5 to 9 h before being removed, during which time they typically laid 200 to 300 eggs. The plates were then further seeded with an additional 20 to 50 µL of heat-killed OP50, then wrapped with Parafilm and incubated at 25.5 °C for 60 to 72 h. Dauers were then picked from this plate for analysis.

### In vivo imaging and analysis of gut granule autofluorescence dynamics

In vivo imaging and analysis were performed with a Zeiss Imager Z2 microscope equipped with an Apotome 2 and Axiocam 506 mono using Zen 2 Blue software. Worms were partially immobilized with 1 mM levamisole in S-basal medium (Stiernagle 2006) and mounted on 2 or 5% agarose pads on microscope slides and observed with a 100× objective.

The excitation wavelength (filter) was 450 to 490 nm, and the emission filter was 500 to 550 nm. If no green flash phenomenon was observed within 180 to 300 s of continuous light exposure, the worm or intestinal region was characterized as not having the phenomenon. The observation was either done through live observation through the eyepiece of the microscope or by reviewing the acquired image series. For the DAPI channel experiments, the excitation wavelength (filter) was 370 to 410 nm, and the emission filter was 430 to 470 nm. Image series (or video) were acquired at a rate of about 5 frames per second, except for the experiments with L4 stage *C. elegans* larvae, which were acquired at a rate of about 6.5 frames per second.

Hermaphrodite or female adult and late larvae (L4/J4) animals were selected based on vulva and gonad morphology. In *C. elegans*, adult animals were young adults developed from L4 animals selected on the previous day. First larval (juvenile) stage (L1/J1) animals of *S. hermaphroditum*, *O. tipulae*, *P. redivivus*, and *Panagrolaimus* sp. PS1159, and second juvenile stage (J2) animals of *P. pacificus* (*P. pacificus* hatches as J2 (Fürst von Lieven 2005)) were selected based on size, general morphology, and gonad morphology (4-cell gonad). In *C. elegans*, first larval stage (L1) animals were obtained by hatching eggs (embryos) obtained by dissolving gravid hermaphrodites (using a mixture of bleach and sodium hydroxide) overnight in M9 buffer, similar to what was described in (Stiernagle 2006). Second larval stage (L2) *C. elegans* were obtained by allowing the synchronized L1 to develop for 24 h at 20 °C on NGM plates seeded with OP50.

### Sample preparation for ex vivo imaging

L4 stage hermaphrodite worms were transferred to a drop of Iwasaki–Teramoto (I–T) solution (Teramoto and Iwasaki 2006) (136 mM NaCl, 9 mM KCl, 1 mM CaCl_2_, 3 mM MgCl_2_, 77 mM D–glucose, and 5 mM HEPES [pH 7.4]) and immobilized with 1 mM levamisole on a microscopic slide. To expose the intestine for imaging, worms were incised with a pair of 30G 5/8″ needles (PrecisionGlide, BD, Franklin Lakes, NJ) near the anus. For experiments with food added on the slide, a glob of OP50 collected by scraping the lawn off an NGM plate using a cell scraper was transferred into the droplet using a platinum–wire worm pick. A circle was drawn using a PAP pen (RPI) around the solution droplet to avoid spilling, and a piece of cover glass was subsequently mounted on top.

### Confocal microscopy

Confocal time series were acquired using a Fluoview FV3000 Confocal laser scanning biological microscope with a 60×, 1.30 N.A. silicone oil objective (Olympus). Frame rate was calibrated to 1 Hz by adjusting the size of the imaging window and the line averaging multiplier. Five minutes of time-lapse images were acquired for each sample. All image processing and analyses were done using ImageJ (National Institutes of Health). The transmitted light channel was illuminated simultaneously with the imaging laser (473 nm) for positional imaging. Panel a1 in Fig. 4, as well as panels b2, d2, f2, and g in Fig. 3, were pseudo-colored with mpl-inferno LUT in FIJI (Schindelin et al. 2012).

### Confocal imaging data processing

ROIs of granules were manually selected in FIJI for all data. For Fig. 2g to h, ROIs of peri-granule “cloud” were defined based on the identification of the fluorescent pattern of green flash events, as the surrounding rings that do not overlap with the granule. The zero points of the time series data were set at the phenomenon onsets of each granule and pooled into metadata.

ROI selection: ROIs were defined based on visual identification of granules in the intestine region, and the fluorescence normalization baseline was set accordingly as the average of the period prior to the changes in fluorescence. Standard deviation envelopes were visualized in the time series. For Fig. 3h, the number of gut granule green flash events was normalized as per minute ∗ 10^4^ µm^2^ of the intestinal surface area from single optical sections, which are manually delineated (Fig. 3g). Nonparametric Mann–Whitney *U* test was used for comparison between WT (wild-type) food vs no-food, and WT food vs *glo-1(*lf*)*.

### Light stimulus and locomotion analysis

Locomotion analysis and animal size measurement were done similarly to what was described in Tan et al. (2022) and Wong et al. (2019) with adaptations. Briefly, at least an hour before the experiment, young adult worms (1 d post–L4) were transferred to NGM plates freshly seeded with 50 µL of OP50. Five worms were transferred per plate. The recording and tracking of the worms were performed using WormLab (MBF Bioscience, Williston, VT) equipment and software. The camera was a Nikon AF Micro 60/2.8D with zoom magnification. Each plate was recorded for 2 min without stimulus followed by 5 min of blue light stimulus. The raw data from WormLab was then manually processed by matching the tracking data with the recorded video. The average speed shown in Fig. 5 was based on the average of the absolute value of the locomotion data (recorded at the frequency of 7.5 Hz) collected at the time span of 2 s (15 frames).

### Data and statistical analysis

Data were plotted as mean ± SEM in Fig. 3 and as mean ± SD in Supplementary Fig. 1. In Fig. 2i, as the raw data failed to pass all normality tests (Kolmogorov–Smirnov test, Shapiro–Wilk test, D’Agostino and Pearson test), the data were plotted as median ± 95% CI instead. Mann–Whitney *U* test was used in Fig. 2 and Student's *t*-test was used in Supplementary Fig. 1.

## Results

### Autofluorescent lysosome-related organelles in the intestine of *C. elegans* displayed dynamic changes in fluorescent intensities when exposed to blue light

**Fig. 1.**
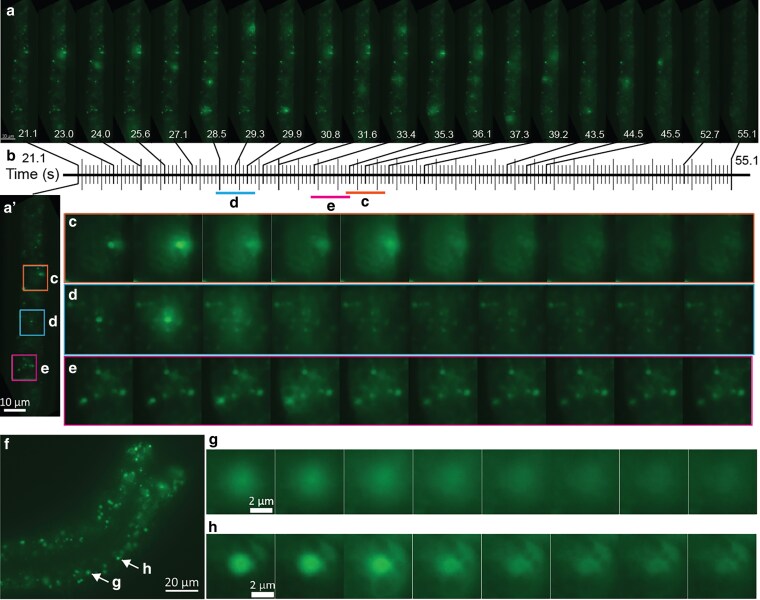
Autofluorescent granules in intestinal cells displayed dynamic changes in fluorescence intensities. a to e) Dynamic fluorescence intensities change in the intestinal granules of a dauer stage larva. a) Fluorescence images (eGFP channel) of the intestine of a dauer stage larva of wild-type (N2) *C. elegans* at different time points during the analysis. The values represented are seconds and start (from 0) with the initiation of live imaging. The complete series of images is presented in Supplementary Video 1. a′) An enlarged image of A at time 0, with sections represented in (c to e) indicated with boxes. Due to the movement of the animal, the positions of the granules were not exactly the same as in (c to e). b) A timeline positioning the images of (a to e). Each vertical line represents an individual time point with an image taken. The images were acquired at a rate of ∼5 frames per second. c to e) Enlargement of sections of the intestine shown in a), focusing on individual granules at the time of rapid intensity changes over periods of ∼1.8 s. In each case, the dissipation of the granules’ autofluorescence was preceded by an increase in intensity (second panel in c and d, third in e) and then a “diffusing” cloud that increased the fluorescent intensities of the surrounding areas. In c), fluorescence intensity changes in the fifth panel may be due to a second granule undergoing the process in a different focal plane. In e), the granule can still be clearly observed after the event, albeit with decreased fluorescence intensities. f to h) Fluorescent dynamics in intestinal granules of an L4 stage larva. f) Fluorescent images (eGFP channel) of the intestine of an L4 stage larva of wild-type (N2) *C. elegans*. g, h) Enlargement of sections of the intestine shown in f), focusing on individual granules at the time of the dynamic changes over periods of about 1.1 s. The complete series of images can be found in Supplementary Video 2. Scale bar: (a, a′): 10 µm; f): 20 µm; (g, h): 2 µm.

When observed with the GFP channel, the autofluorescent granules in the intestine of *C. elegans* can undergo dynamic changes in fluorescence intensity (Fig. 1; Supplementary Videos 1 and 2). Specifically, there is a sharp increase in fluorescence intensity in the granules, followed by a quick dissipation and diffusion into the surrounding area (Fig. 1). This “flash” behavior of the autofluorescent granules can be observed in some but not all developmental stages. We observed the events in the stress-resistant dauer stages larvae (15 of 20 worms) (Fig. 1a to e; Supplementary Video 1), the second stage (L2) larvae (5 of 5 worms), and the fourth stage (L4) larvae (19 of 20 worms) (Fig. 1f to h; Supplementary Video 2), but not in the first stage (L1) larvae (0 of at least 5 worms) and adult animals (0 of 5 worms).

We reasoned that these autofluorescent granules are lysosome-related organelles known as gut granules (Laufer et al. 1980). We examined *glo–1(lf)* worms deficient in gut granule biogenesis and observed neither these granules nor the flash phenomenon in the *glo-1(lf)* worms (L4, 0 of 11 worms) (Hermann et al. 2005), suggesting that the granules with the flash phenomenon are gut granules. We therefore refer to the phenomenon as “gut granule green flash.”

We hypothesized that the phenomenon was induced by the blue light used for excitation since it only occurs after the start of the imaging process (Supplementary Videos 1 and 2). Indeed, increasing the intensity of the blue light significantly shortens the amount of time it takes for the flash phenomenon to occur in the intestine of wild-type dauer stage larvae—45.4 s (range: 41 to 54, n = 5), when the intensity was set at 10% maximum; but down to 15.2 s (range: 13 to 19, n = 5) and 12 s (range: 9 to 14, n = 5), when the intensity was set at 50% and 100%, respectively.

### At least 2 types of fluorophores are likely present in the gut granules with distinct fluorescence dynamics

**Fig. 2.**
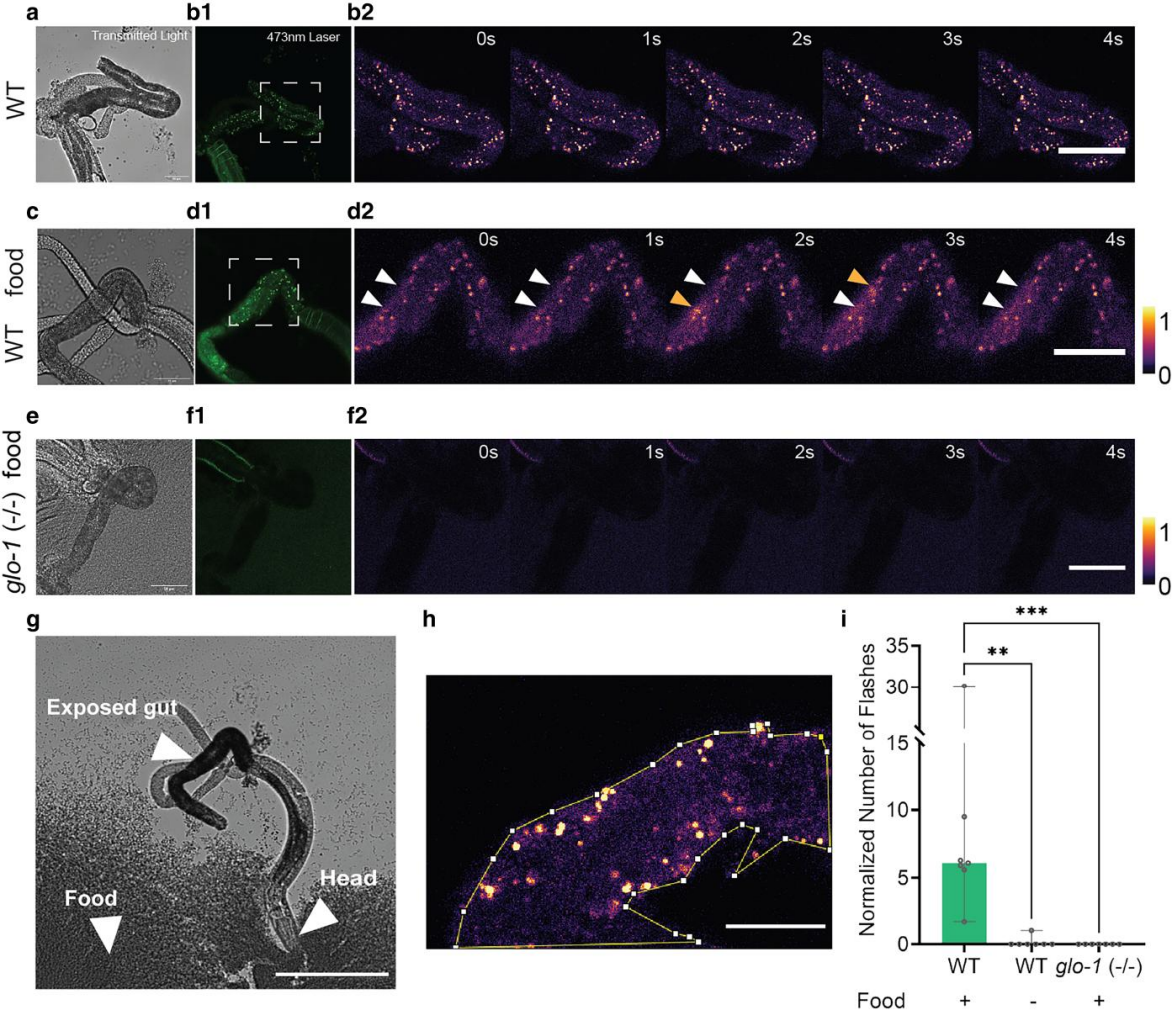
Gut granule green flash is correlated with food availability in the ex vivo experimental setting. The field of view was illuminated and recorded with transmitted light (a, c, e), epifluorescence (b1, d1, f1), and 473 nm laser illumination (Ex: 473 nm; Em: 500 to 550) (b2, d2, f2). In (b2, d2, f2), 5-s clips of time series with 473 nm laser illumination were pseudo-colored and shown. a, b) In wild-type worms not provided with food on the slide, the phenomenon was rarely observed. c, d) In wild-type worms provided with food on the slide, the occurrences of the phenomenon increased dramatically. Orange arrowheads (the lower arrowhead in 1s and the upper arrowhead in 2s) in d2) indicate the positions of gut granule green flash events. White arrowheads indicate the positions of those events before and after the flash. e, f) No gut granule fluorescence and no events were detected in *glo-1(lf)* worms, even in the presence of food. g) An example of the ex vivo experimental preparation. L4 stage nematodes were carefully incised near the anus to expose the gut. In the “with food” conditions, nematodes were positioned to embed their heads in food. h, i) Gut granule green flash events were manually identified and analyzed. To normalize the number of events for comparison, the counts for each sample were temporally averaged in the area of exposed intestine within the field of view, as shown in h) (details in Materials and methods). i) Normalized data from all conditions were plotted (N = 6 to 7), and each group was compared with the wild-type (WT) + food group (**P < 0.01, ***P < 0.001, Mann–Whitney *U* test). The vertical bar indicates the data range. Scale bar: (a to f): 50 µm; g): 200 µm; h): 20 µm.

To investigate the dynamics of gut granule green flash, we extruded the intestines of L4 stage hermaphrodites by cutting the animal open posteriorly near the anus (Fig. 2g) and imaged the intestine using scanning confocal microscopy. Under this ex vivo experimental setting, we were able to observe and characterize the fluorescence dynamics with increased resolution. However, in contrast to the in vivo setting, we found gut granule green flash to be rare under this condition. Part of the difference could be due to the intensities of the stimulating light. Since the primary function of the intestine is food digestion, we reason that autofluorescent granules’ fluorescence flashes could be associated with nutrient uptake. In *C. elegans*, food availability significantly influences the rhythmic defecation cycle (Thomas 1990), controlled by calcium oscillations in the intestinal cells (Dal Santo et al. 1999). To test whether gut granule green flash is associated with food availability, we provided food (*Escherichia coli*  OP50) to the experimental animal on microscopic slides (Fig. 2g). We found that in worms with a significant amount of food near the head region, the occurrence of the gut granule green flash increased dramatically (Fig. 2a to d; Supplementary Video 3). All 7 recordings of worms provided with food displayed the phenomenon (Fig. 2c, d, and i), while only 1 of 7 without food did, and at a low frequency (Fig. 2a, b, and i). It is unclear why the autofluorescent granules’ fluorescence flash phenomenon in the ex vivo setting was strongly correlated with food availability, but it may imply that gut granule green flash could be a part of or a consequence of a metabolic or signaling process in food intake. A possible explanation for the difference in food dependency in the in vivo and ex vivo settings is that only in the ex vivo setting, with no provision of food, were the intestinal cells removed from the usual content of the intestinal lumen. In subsequent experiments in which we examined gut granules’ fluorescence flash in ex vivo intestines, we provided food to increase the frequency of the flash.

To further test whether these autofluorescent granules that flash are gut granules, we analyzed the ex vivo intestines of *glo-1(*lf*)* mutant animals. *glo-1(*lf*)* animals displayed a large reduction in the number of autofluorescent granules in the intestine, similar to what was described by Hermann et al. (2005), and also completely eliminated detectable flashes (Fig. 2e, f, and i). None of the 7 *glo-1(*lf*)* animals displayed food-induced flashes of fluorescence, as seen in all 7 wild-type animals (Fig. 2i), suggesting that the autofluorescent granules that flash are gut granules.

**Fig. 3.**
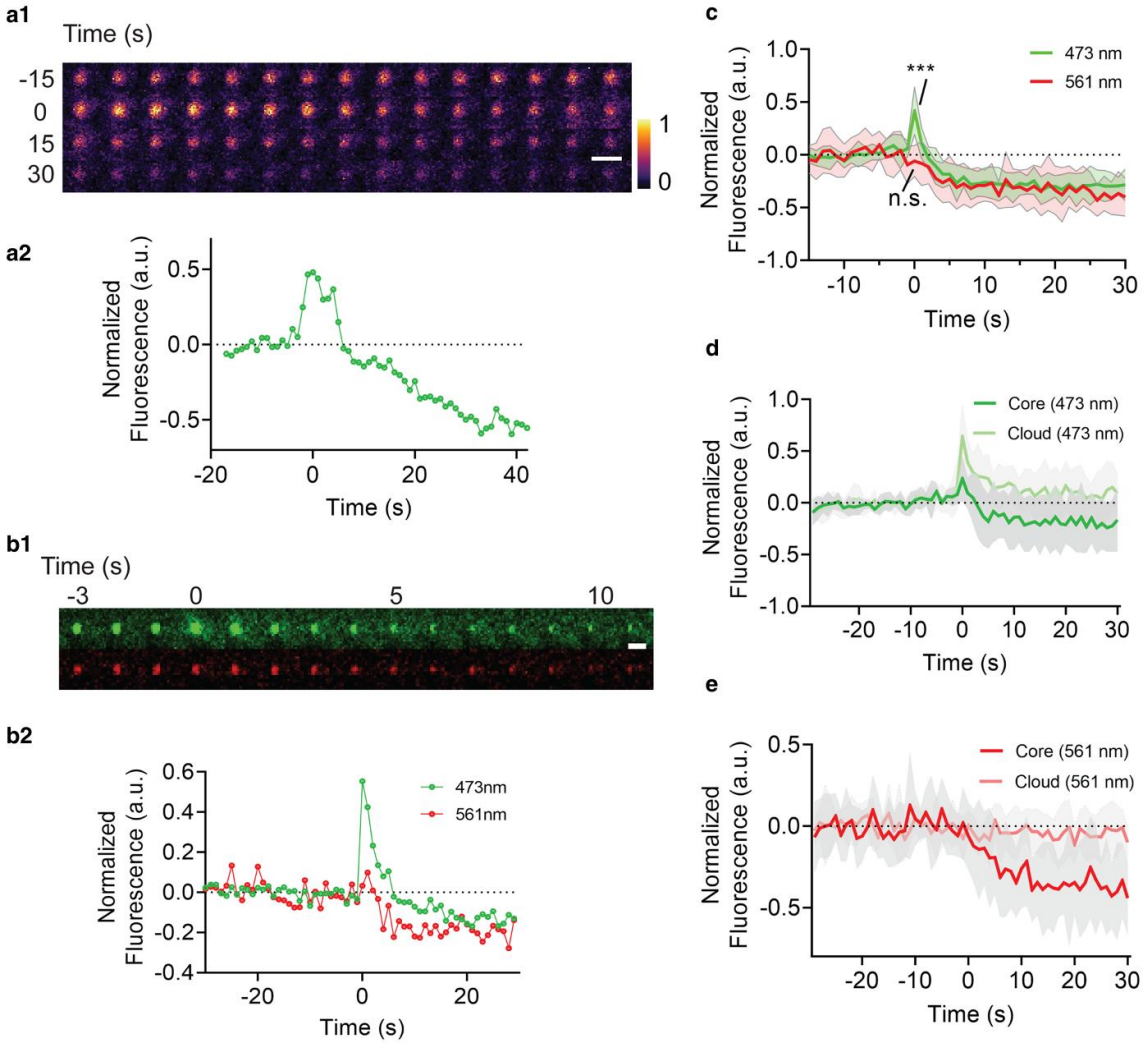
The dynamics of gut granule green flash. a) The green fluorescence in the granule increases and falls sharply. Representative example of changes in green fluorescence during gut granule green flash. a1) A 1-min pseudo-color time series of a gut granule green flash event. Imaging rate: 1 Hz. The time axis is zeroed at the onset of changes in fluorescence (see Materials and methods). a2) Normalized green fluorescence intensity plot of the same granule shown in a1). b, c) The green fluorescence in the granule increases and falls sharply, while the red lacks the initial sharp increase. b) A representative example of 2-color time-lapse imaging b1) and normalized quantifications b2). In (a2 and b2), the fluorescence intensities of each granule are normalized by the baseline fluorescence calculated as the mean of fluorescence in the ROIs prior to the changes in fluorescence. c) The average fluorescence intensity of all recorded green flash events in 1 sample. The green fluorescence shows a distinct peak after time 0, while the red fluorescence does not. d, e) Gut granule flashes. Two-color imaging of all recorded gut granule green flash events in a representative sample. The normalized fluorescence of each data point at time 0 in the green channel is compared to baseline (normalized fluorescence = 0) and shows a statistically significant difference (***), while a parallel comparison between red channel data point vs baseline does not show a significant difference (n.s.). d, e) The granular area (“core”), as well as the surrounding ring area that presents diffusion of fluorescence (“cloud”), were measured separately in both 473 nm (Ex: 473 nm; Em: 500 to 550) d) and 561 nm (Ex: 561 nm; Em: 580 to 680) e) illumination. d) The intensity increase in green fluorescence is not only limited to the original “core” area but also to the surrounding “cloud.” e) No intensity increase was observed with the red fluorescence, neither in the “core” nor the “cloud.” Raw images for analysis were identical to c), except a longer pre-onset timeframe was selected for normalization. In (c to e), pooled values are presented as mean ± SD (N = 11 in d, N = 8 in d and e). The time axis is zeroed at the onset of changes in fluorescence. Scale bar: 5 µm.

When wild-type worms under the ex vivo setting were provided with food, we observed that the green fluorescence, illuminated with the 473 nm laser (Ex: 473 nm; Em: 500 to 550) of the confocal microscope, rose sharply and significantly (Fig. 3a to c; Supplementary Video 3). The intensity increase is not only limited to the original “core” area (the granule proper, as identified by the high level of fluorescence prior to the event) but also to the surrounding areas (which we refer to as the “cloud”; details of how the area is identified are in Materials and methods; Fig. 3d). The fluorescence intensity in both the “core” and the “cloud” subsequently dropped off, with the intensity at the “core” decreasing to a level lower than baseline, and the intensity in the “clouds” recovered to the baseline (Fig. 3d). Nevertheless, when illuminated with the 561 nm laser (Ex: 561 nm; Em: 580 to 680), the emitted red fluorescence lacked the initial sharp increase observed in green fluorescence but fell concurrently (Fig. 3e). We estimated that the rapid changes in fluorescence intensity occur within 10 s. The differences observed in the dynamics of green and red fluorescence intensities suggest that they likely represent 2 distinct types of fluorophores. It is also possible, however, that they represent a single fluorophore with 2 distinct transitions.

One possible mechanism behind the green flash phenomenon is the breakdown of gut granule membranes induced by the energy carried by the blue light from the GFP channel. We, therefore, explore whether exposing the worm under the shorter wavelength DAPI channel would give a similar result. We found that continuous exposure to DAPI channel light turned the intestine/worm blue (Supplementary Video 4), but a similar fluorescence flash phenomenon was not observed (tested in the intestine of wild-type dauer stage larvae, with 10%, 50%, and 100% intensity as with the previous experiment with GFP channel; at least 5 animals were tested in each setting). One of the fluorophores residing in the gut granules has been identified as anthranilic acid (Babu 1974; Coburn et al. 2013), which has been associated with the increase in the blue fluorescence near the death of the animals (“death fluorescence”). Although the fluorescence changes described in this study are distinct both spatially and temporally from the death fluorescence reported, it is still possible that the breakdown of gut granule membranes causes both.

In spite of its lack of an obvious light-sensing organ, *C. elegans* exhibits negative phototaxis (Edwards et al. 2008; Ward et al. 2008; Bhatla and Horvitz 2015). Given our finding that gut granules may be light sensitive, we wondered whether their presence is associated with the animal's light response. We found that exposure of worms to blue light increases the locomotion of *glo-1* mutant worms that are defective in gut granule biogenesis to a similar extent as wild-type worms (Supplementary Fig. 1). These results suggest that gut granules are not involved in the worms’ light avoidance behavior.

### Autofluorescent granules are widespread in nematodes

To explore whether the biology behind the green flash phenomenon is common in nematodes, we first examined whether gut granule-like autofluorescent granules are present across different nematode species.

Gut granules in *C. elegans* are considered to be rhabditin granules (Laufer et al. 1980). Birefringent granules in “*Rhabditis*” species were first described by Maupas (1900), with the term “rhabditin” used to describe the organic material (crystal) that was the likely source of the birefringence (Cobb 1914; Chitwood and Chitwood 1950). Such birefringent granules are common in “*Rhabditis*” species (including the current nematode genus such as *Caenorhabditis*, *Oscheius*, and *Pelodera*) (Maupas 1900; Cobb 1914; Chitwood and Chitwood 1950; Thomas and Quastler 1950; Bossinger and Schierenberg 1992), but have also been reported in an evolutionary distant nematode, *Spiroxys contortus* (Hedrick 1935 ), and autofluorescence has been reported to be widespread in nematode intestines in the form of globules (Forge and Macguidwin 1989).

We examine the intestinal autofluorescence of 5 other species of nematodes that are evolutionarily divergent but have established laboratory culture methods. These are *O. tipulae* (Lam and Webster 1971 ); *P. pacificus* (Sommer et al. 1996); *S. hermaphroditum* (Stock et al. 2004); *P. redivivus* (Linnaeus), and a species of the *Panagrolaimus* genus (*Panagrolaimus* sp. PS1159) (Sommer and Sternberg 1996). With *C. elegans* included, we examined 3 members each of Clades IV (*Steinernema*, *Panagrellus*, *Panagrolaimus*) and V (*Caenorhabditis*, *Oscheius*, *Pristionchus*) nematodes based on the 5 major clade classification of Blaxter et al. (1998).

**Fig. 4.**
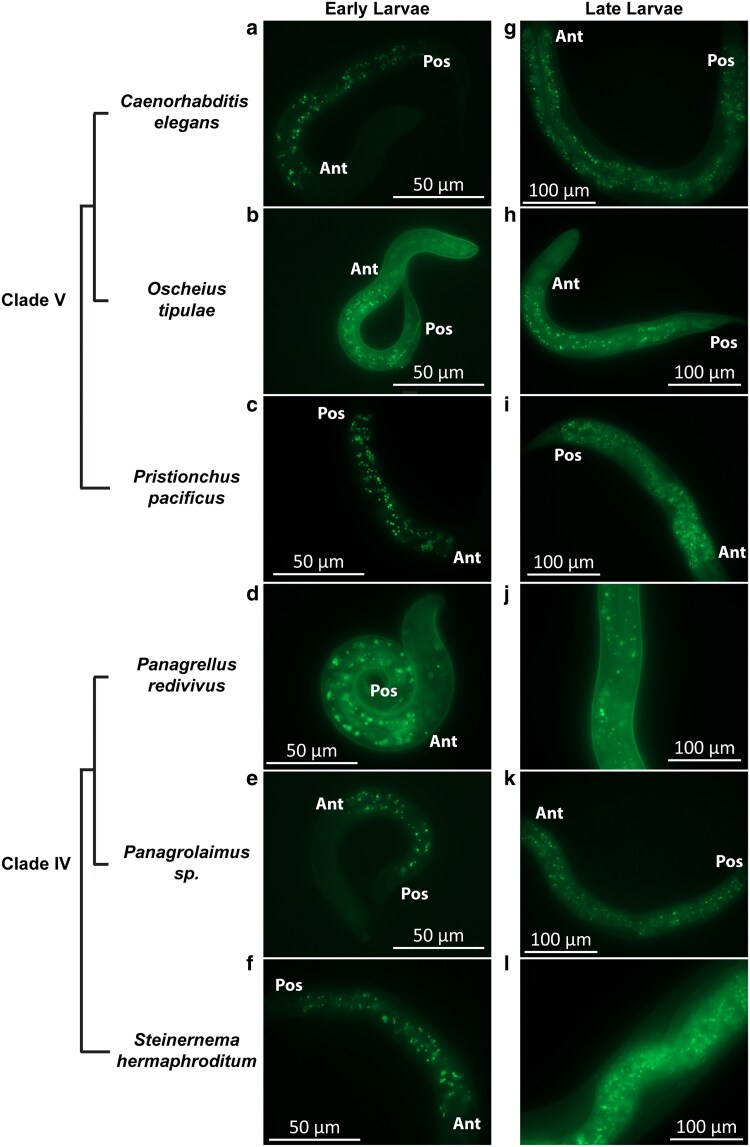
Autofluorescent granules are widespread in nematodes. Epifluorescence images (eGFP channel) of (a to f) early larvae. g to l) Late larvae. a) L1 *Caenorhabditis elegans*. b) L1 *Oscheius tipulae*. c) J2 *Pristionchus pacificus*. d) L1 *Panagrellus redivivus*. e) L1 *Panagrolaimus* sp. PS1159. f) J1 *Steinernema hermaphroditum*. g) L4 *C. elegans*. h) L4 *O. tipulae*. i) J4 *P. pacificus*. j) L4 *P. redivivus*. k) L4 *Panagrolaimus* sp. PS1159. l) J4 *S. hermaphroditum*. “Ant” indicates the anterior end of the intestine; “Pos” indicates the posterior end of the intestine. Scale bar: (a to f): 50 µm; (g to l): 100 µm.

**Fig. 5.**
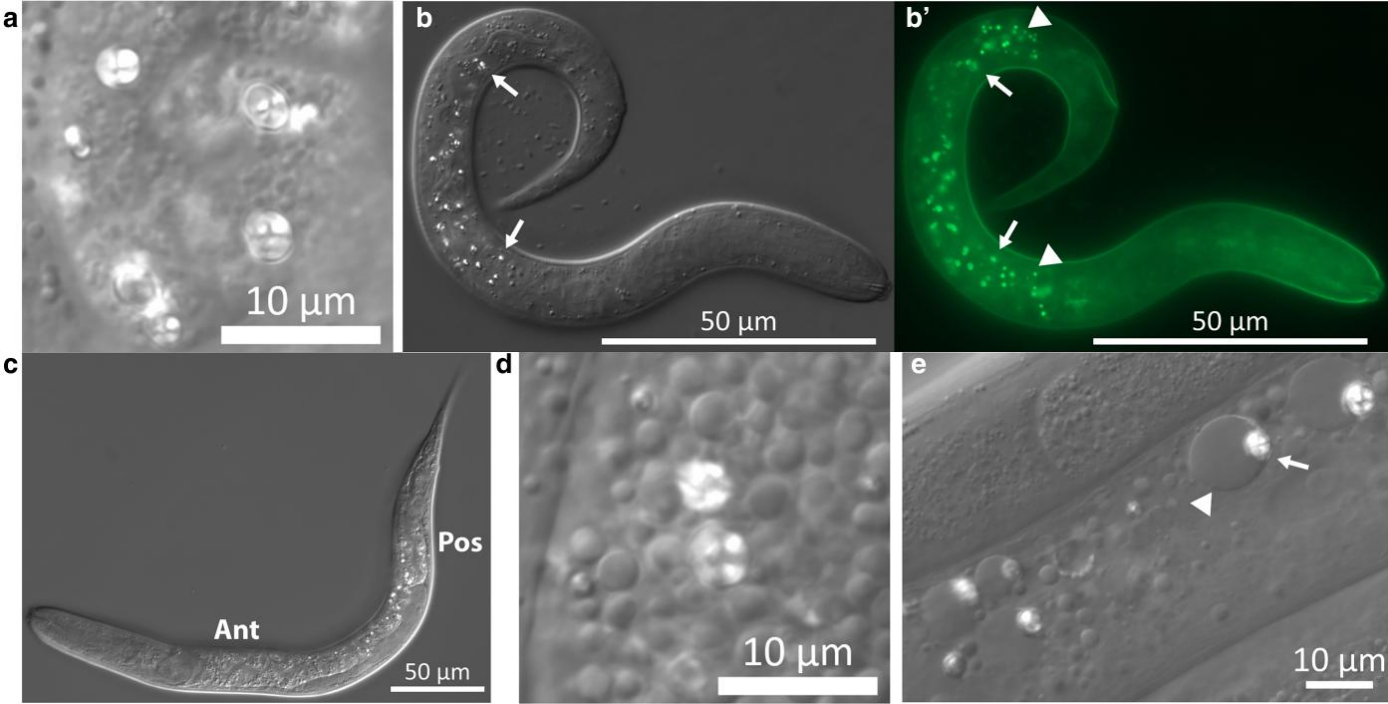
Birefringent granules in nematode intestine. Birefringent granules can be easily observed with Nomarski microscopy (DIC) in the intestine cells of *O. tipulae* (a to c) and *P. redivivus* (d, e). a) Birefringent granules of *O. tipulae*. Some granules have a Maltese cross appearance. The image is from an adult hermaphrodite. b, b′) A L1 *O. tipulae* larvae imaged with DIC b) or eGFP channel b′). The birefringent granules are also autofluorescent, but autofluorescent granules may not always be birefringent. Arrows point to 2 granules that are both birefringent and autofluorescent. Arrowheads point to 2 granules that are autofluorescent but not birefringent. Most birefringent granules are in the mid-gut, while most of the autofluorescent granules in both ends of the intestine are not birefringent. The head of the animal is toward the right. c) A mid-larvae (∼L3) stage *O. tipulae* imaged with DIC. The distribution of the birefringent granules shows a strong bias toward the posterior half of the intestine. “Ant” indicates the anterior end of the intestine; “Pos” indicates the posterior end of the intestine. d) Birefringent granules of *P. redivivus*. Some granules have a Maltese cross appearance. The image is from an L4 female. e) Birefringent granules (arrows) in the intestine of adult *P. redivivus* are often associated with larger non-birefringent granules (arrowhead). The granules are closely associated, as can be seen in Supplementary Video 5. Scale bar: (a, d, e): 10 µm; (b, c): 50 µm.

In all species examined, we found autofluorescent granules to be present at least in the early and late larval stages (Fig. 4), with observable differences in distribution and morphology. In *O. tipulae* and *P. redivivus*, some of the granules are strikingly birefringent and can easily be observed with Nomarski differential interference contrast microscopy (DIC) (Fig. 5). At higher magnification, at least some of these birefringent granules have a Maltese cross appearance (Fig. 5a and d), similar to what has been described by Cobb (1914). The birefringent granules are also autofluorescent (Fig. 5b), but at least under Nomarski microscopy (DIC), not all autofluorescent granules are birefringent.

**Fig. 6.**
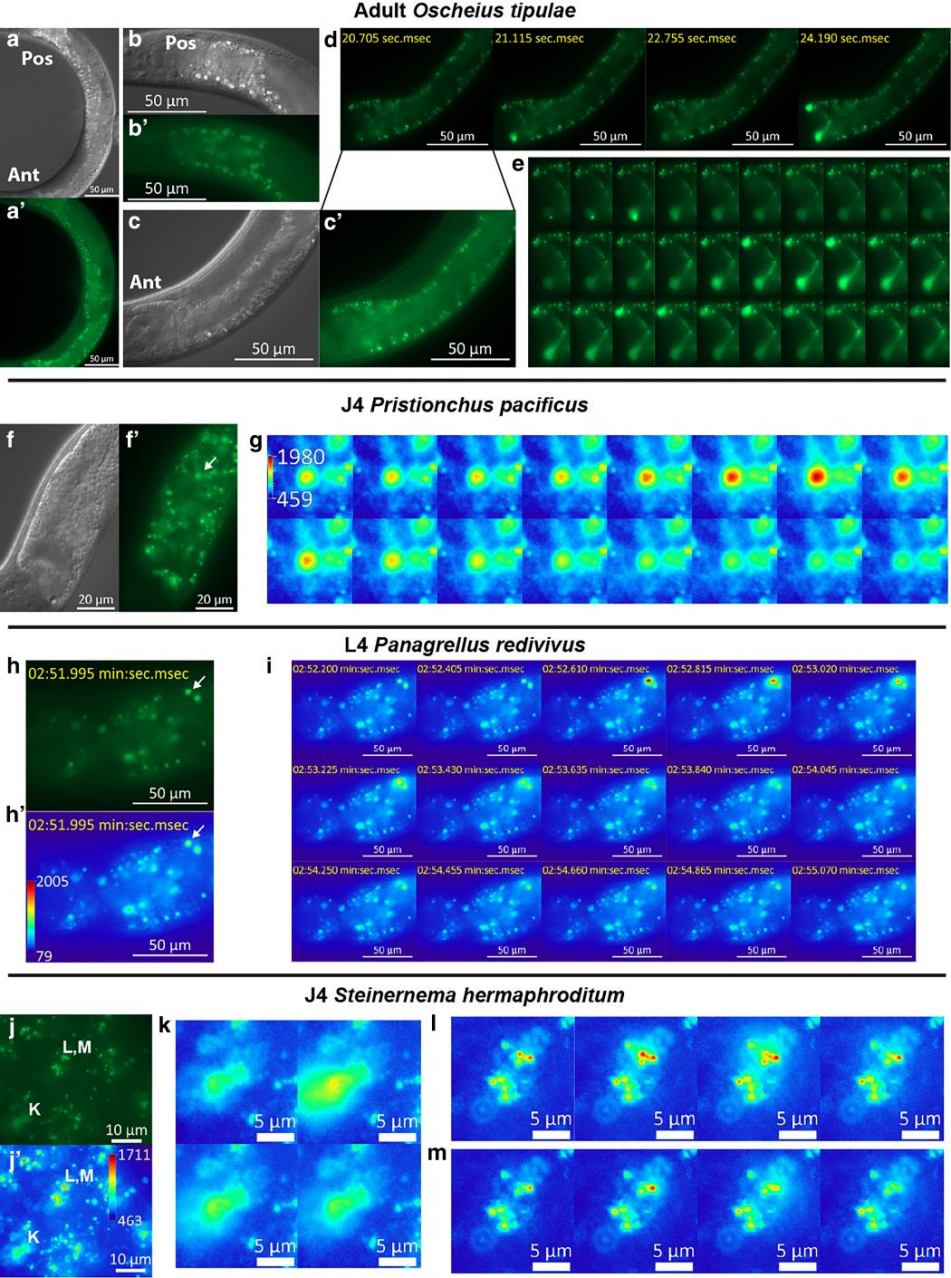
The green flash phenomenon of autofluorescent granules in intestinal cells is shared among nematodes. a to e) Adult hermaphrodite *O. tipulae*. Scale bar: 50 µm a) Nomarski microscopy (DIC) and a′) epifluorescence (eGFP) image of the worm's intestine. “Ant” indicates the anterior end of the intestine; “Pos” indicates the posterior end of the intestine. The distribution of the birefringent granules has a strong posterior bias. b, b′) The enlarged DIC b) and eGFP b′) image of the posterior end of the intestine. No obvious green flash phenomenon was observed in this region (Supplementary Video 6). c, c′) The enlarged DIC c) and eGFP c′) image of the anterior end of the intestine. The autofluorescent granules at the anterior end are not birefringent under DIC and are often somewhat smaller in size. d) Fluorescence images (eGFP channel) of the anterior end of the intestine at different time points during the analysis. Values represented are seconds and milliseconds and start (from 0) with the initiation of live imaging. The phenomenon is most commonly observed in the first intestinal cell ring. The complete series of images can be found in Supplementary Video 7. e) eGFP time series focusing on the first intestinal cell ring, covering largely the same period as shown in d) (start at the same frame), the images were acquired at a rate of ∼5 frames per second. f, g) J4 hermaphrodite *P. pacificus*. f, f′) Nomarski microscopy (DIC) f) and epifluorescence (eGFP) f′) image of the anterior intestine. The arrow points to the gut granule detailed in the time series k). Scale bar: 20 µm. k) eGFP (pseudo-colored-heat map) time series of the green flash of the granule indicated by the arrow in f′), the images were acquired at a rate of ∼5 frames per second. The number on the vertical bar indicates the range of the color assignment of the heat map. The complete series of images can be found in Supplementary Video 8. h, i) epifluorescence (eGFP) images of the anterior intestine of a J4 female *P. redivivus*. Scale bar: 20 µm. h, h′) Pseudo-colored-green h) and pseudo-colored-heat map h′). The arrow points to a gut granule that flashes, as shown in the time series i). Scale bar: 50 µm. The number on the vertical bar h′) indicates the range of the color assignment of the heat map. i) Time series: The images were acquired at a rate of ∼5 frames per second. Scale bar: 50 µm. j to m) Epifluorescence (eGFP) images of a section of the anterior intestine of a J4 hermaphrodite *S. hermaphroditum*. Pseudo-colored-green j) and pseudo-colored-heat map j′). The labels “L, M” and “K” indicate the corresponding enlarged time series images in k) and “L, M” (L and M are 2 time series of the same region). Scale bar: 10 µm. The number on the vertical bar j′) indicates the range of the color assignment of the heat map. Scale bar: 5 µm. The complete series of images with lower resolution can be found in Supplementary Video 9.

In *O. tipulae*, the pattern in which these birefringent granules are distributed in the intestine varies by stage (Fig. 5b and c). In J1 larvae, they are predominantly mid-gut (Fig. 5b), but by later stages, a clear spatial difference emerges (Fig. 5c). Specifically, the autofluorescent granules at both ends of the intestine remain largely non-birefringent throughout development (Figs. 5b and 6a to c, Supplementary Fig. 2). The birefringent granules and non-birefringent autofluorescent granules might represent 2 distinct groups of LROs.

In adult *P. redivivus*, the birefringent granules are often clearly associated with a larger non-birefringent granule (Fig. 5e, Supplementary Video 5). The association bears a resemblance to the bilobed structure in the gut granules of *C. elegans* observed under excessive and deficient zinc conditions (Roh et al. 2012; Mendoza et al. 2024), with the larger non-birefringent granule resembling the expansion compartment observed.

### Gut granule green flash is common in nematodes

After establishing that autofluorescent granules similar to gut granules are common in nematodes, we explored whether this is also true for the “green flash” phenomenon. Of the 6 species (including *C. elegans*) tested, we were able to reliably observe and record the phenomenon in all but *Panagrolaimus* sp. PS1159 (Table 1, Fig. 6, Supplementary 2, Supplementary Videos 7 to 11). In all species with the green flash phenomenon, it could always be observed in at least some of the larvae/juvenile stage 4 (L4/J4) animals (Late larvae) (Fig. 6f to m, Supplementary 2a-b, Supplementary Videos 8 to 10). This commonality persists despite the large difference in size, morphology, and lifestyle of these species, suggesting that there might be some common biological process that occurs at this stage of development. In addition to the L2 and dauer stage *C. elegans* (Fig. 1a to e, Supplementary Video 1), we observed the phenomenon in the J1 animal of *S. hermaphroditum* and the adult (hermaphrodite) animal of *O. tipulae* (Fig. 6a to e, Supplementary Fig. 2c to e, Supplementary Videos 7 and 11), so it is not exclusive to late larval stages (Table 1).

**Table 1.** Developmental stages in which gut granule green flash can be observed.

Species							
Stages		*Caenorhabditis elegans*	*Oscheius tipulae*	*Pristionchus pacificus*	*Panagrellus redivivus*	*Panagrolaimus* sp. PS1159	*Steinernema hermaphroditum*
E. Larvae	L1/J1	X	Δ	NA	Δ	X	O
	L2/J2	O		X			
Dauer		O					
L. Larvae	L4/J4	O	O	O	O	X	O
Adult		X	O	X	X	X	X

O, yes; Δ, rarely; X, never; NA, not applicable; Empty, no sufficient data; E, early; L, late.

In both late larvae and adult animals, we also observed that there is a strong anterior bias for the green flash phenomenon. When observed, it occurred at the anterior end of the intestine, but rarely at the posterior end. For example, in both J4 hermaphrodite *S. hermaphroditum*, and adult hermaphrodite *O. tipulae*, green flash can be induced at the anterior end of the intestine in all animals (6 of 6 each) with the phenomenon (Fig. 6c to e and j to m, Supplementary Videos 7 and 9), but none at the posterior end (Fig. 6b, Supplementary Video 6); in J4 hermaphrodite *O. tipulae*, green flash can be induced at the anterior end of the intestine in all 10 animals (Supplementary Fig. 2a and b, Supplementary Video 10), but only weakly at the very end of the posterior end in 4 of 10 animals. At least in *O. tipulae*, the phenomenon also occurs predominantly at the ends of the intestine, with the strongest intensity observed at the first anterior intestinal cell ring (Fig. 6d and e, Supplementary 2a and b, Supplementary Videos 7 and 10). It is worth noting that the distribution pattern of the flashable autofluorescent granules in *O. tipulae* (anterior-biased, concentrated at the ends of the intestine) is opposite to the autofluorescent granules that are also birefringent, as previously noted (posterior-biased, concentrated at the middle section of the intestine). In both *O. tipulae* and *P. redivivus*, we did not observe obvious green flash events in the birefringent granules.

Overall, we conclude that the population of autofluorescent granules in nematodes comprises 2 or more subpopulations with distinctive differences in biochemical properties that undergo significant changes during development, and that the green flash phenomenon is one of the assays that could be used for identifying these populations.

## Discussion

*C. elegans* has long been known to be autofluorescent (Babu 1974), with most of the autofluorescence coming from the gut granules (Clokey and Jacobson 1986). Particular interest has been paid to the increasing level of autofluorescence as the worms age or are stressed (Klass 1977; Davis et al. 1982; Clokey and Jacobson 1986; Forge and Macguidwin 1989; Pincus et al. 2016; Hajdu et al. 2023). However, the fluorophores responsible for gut granule autofluorescence remain largely undefined. One of the fluorophores emitting blue fluorescence has been identified as anthranilic acid (Babu 1974; Coburn et al. 2013), and changes in the blue fluorescence occur near the death of the animals (*d*eath *f*luorescence) have been described (Coburn et al. 2013; Pincus et al. 2016). Both green and red fluorescence also increase with age, and the green fluorescence intensifies near the death of the animals (Coburn et al. 2013; Pincus et al. 2016). It is important to note that these fluorescence changes are distinct from the gut granule green flash phenomenon that we are describing in this research, both spatially and temporally.

In *C. elegans*, gut granules have been shown to have both metabolic and signaling functions. These lysosome-related organelles (LROs) are a site for fat, cholesterol, and heme storage (Schroeder et al. 2007; Lee et al. 2015; Sun et al. 2022) as well as a storage and sequestering site for micronutrient metals (Roh et al. 2012; Chun et al. 2017). Gut granules are also the site of biosynthesis for secondary metabolites, including the signaling molecules ascarosides (Le et al. 2020). Recently, they have also been suggested to be involved in the responses to other forms of stress and in the resistance to pathogens (Hajdu et al. 2023). They are also structurally more complex than a simple sphere with a lipid bilayer. The studies of Roh et al. (2012) and Mendoza et al. (2024) show that gut granules are bilobed with 2 compartments, which they describe as an acidified compartment and an expendable compartment. The structure is also not static, with the volume of the expendable compartment changing at least in response to dietary zinc levels (Roh et al. 2012; Mendoza et al. 2024). The mechanism of such a structural rearrangement remains largely uncharacterized. Unexpectedly, in our survey of nematode intestine granules, we discovered a striking similarity in the granules of *P. redivivus* (Fig. 5e), an evolutionary distant nematode, suggesting that such organelle structure is not unique to *C. elegans* and might be common in nematodes and maybe beyond.

In an attempt to develop the intestine of *C. elegans* as a model to study vesicle release, we discovered a novel phenomenon of dynamic autofluorescence in intestinal lysosome-related organelles. When observed with the green fluorescence channel, these autofluorescent granules of the nematode intestine underwent a rapid and dynamic change in intensity, a phenomenon we describe as “gut granule green flash.” Observable changes begin with a sharp increase in fluorescence intensity and end with rapid dissipation and diffusion. Further analysis suggests that at least 2 distinctive fluorophores or fluorophore transitions exist in the gut granules, with only one of them having the “flash” characteristic.

**Fig. 7.**
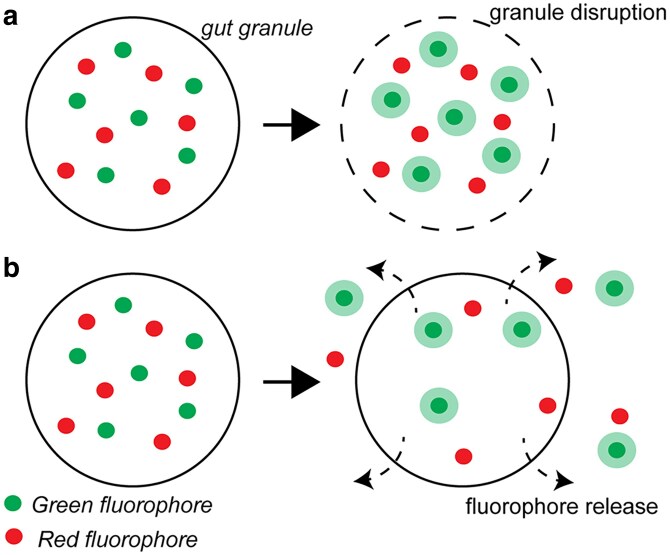
Models for the gut granule green flash phenomenon. In both models, the green fluorophores are pH-sensitive, with fluorescence intensity positively correlated with pH. On the other hand, the red fluorophores are likely pH-neutral. a) In the first model, the fluorescence dynamic is explained by the breakdown of the granule and the release of its constituents. The fluorescence of the green fluorophores increases after breakdown due to the higher pH environment in which the fluorophores now reside. b) In the second model, the fluorescence dynamic is explained by the release of the fluorophores by intact granules. The released green fluorophores increased in fluorescence in the more basic cytosol.

Mechanistically, the green flash phenomenon could be explained either by the breakdown of the granule and the release of 1 or more of its constituents (Model 1, Fig. 7a), or the release of some of the granule constituents by granules that remain intact (Model 2, Fig. 7b). In both models, changes in the green fluorescence intensity may be explained by the green fluorophore responding to differences between the lysosome-related organelles and the cytoplasm. The lumens of the lysosome-related organelles are more acidic than the cytoplasm (Clokey and Jacobson 1986; Kostich et al. 2000; Hermann et al. 2005); if the green fluorophores are pH-sensitive, with fluorescence intensity positively correlated with pH, the green fluorophore would increase in fluorescence upon exiting the granules. On the other hand, the changes in red fluorescence intensity may be explained by the red fluorophore being pH-neutral. The subsequent dissipation and diffusion can also be reasonably explained as the diffusion of both of these molecules. We think Model 1 may be more likely (Fig. 7a), partly because there is some similarity between our observation and that of Luke et al. (2007), in which fluorescently labeled gut granule disruption in osmotic sensitive mutants under hypotonic shock was shown. Also, although the green flash phenomenon is distinct from the death (blue) fluorescence described (Coburn et al. 2013), as the green flash phenomenon is local (surrounding the LRO) and fast, and the death (blue) fluorescence is global and slow, 2 observations from different nematode species lead us to suspect that both could be caused by the breakdown of the gut granules. First, we found that at least in the dauer stage of *C. elegans*, continuous exposure to the shorter wavelength DAPI channel quickly turned the intestine/worm blue (Supplementary Video 4). The change in the blue fluorescence bears some resemblance to the death (blue) fluorescence described. Second, although it is unclear for most of the nematode/stages we analyzed, the gut granule green flash does seem to contribute to the buildup of overall cytostome green fluorescence in the anterior intestine of *O. tipulae* (Supplementary Video 10). We do not exclude Model 2, however, as we noticed that it is not uncommon for residual autofluorescence to remain after the flash event for individual granules (Fig. 1e), and in the ex vivo confocal recorded experiments, we have also observed multiple flash events in some of the granules, both of which argued against the disruption of the granules and support substance release by intact granules as described in Model 2 (Fig. 7b). It is also possible that the event described in both models occurs, but in different granules.

Regardless of the potential mechanisms, we believe that stimulation via blue light is likely the cause, as the phenomenon was usually not observed immediately upon illumination, and that increasing the intensity of the blue light significantly shortens the amount of time it takes for the flash to occur. However, we cannot rule out that gut granule green flash may also be naturally occurring. In the ex vivo setting, the phenomenon was highly associated with the immediate presence of food (Fig. 2), which may suggest that the phenomenon can be a part of or a consequence of a metabolic or signaling process in food intake. One possibility is that this phenomenon is associated with the release of vesicle content from gut granules, and the fluorophores or the content released play metabolic or signaling roles in food uptake. Alternatively, the exposure of the basal face of intestinal cells to bacteria and their metabolites could be a significant stressor to the cells, and it is plausible that the physiological condition in these cells caused the granules to be in a more “flashable” state.

We observed that not all gut granules can be induced to flash in our settings, and their inducibility varies significantly during development. We suspect that this is due to the existence of multiple subgroups of gut granules and that their biology changes significantly in the developmental process. To put the observed phenomenon in a wider context and to rule out that the stage-specific inducibility may be associated with worm size or something specific to *C. elegans*, we explored the green flash phenomenon in other nematode species. We found that the understanding of similar granules in other nematodes is somewhat limited. The first documented description of such granules (described as birefringent, blackish, and opaque with transmitted light) may be that of Maupas (1900), in the same research that also first describes *C. elegans* (*Rhabditis elegans*) as a species. Maupas (1900) suggests the lack of those granules in *C. elegans*. With better microscopy, it is now known that at least some (Laufer et al. 1980 ), if not all (Hermann et al. 2005), gut granules in *C. elegans* are clearly birefringent. Cobb (1914) used the term “rhabditin” to describe the organic material (crystal) that was the likely source of the birefringence he identified in the intestinal cells of *Mesorhabditis monhystera* (*Rhabditis monhystera*), and such “rhabditin granules” have since been found to be common among “*Rhabditis*” species (including the current nematode genus such as *Caenorhabditis* and *Oscheius*) (Maupas 1900; Cobb 1914; Chitwood and Chitwood 1950; Thomas and Quastler 1950). Similar granules have been described outside of the “*Rhabditis*” species; Maupas (1900) described 2 species that now belong to the genus *Pristionchus* with birefringent intestinal granules/corpuscles, and Hedrick (1935) used “rhabditin” to describe a granular substance that the author observed in an evolutionarily distant nematode, *S. contortus*. The autofluorescent nature of the granules was described much later (Laufer et al. 1980; Hermann et al. 2005), and mostly only in *C. elegans*, but Forge and Macguidwin (1989) found autofluorescence “globules” in all 15 diverse species of nematodes they analyzed, and Bossinger and Schierenberg (1992) described the autofluorescence pattern of gut primordium in *Oscheius dolichura* (*Rhabditis dolichura*, also described by (Maupas 1900) to be birefringent) embryos in comparison to that of *C. elegans*. It is likely that, in most of these species, the birefringent or autofluorescent granules/corpuscles/globules correspond to the gut granules of *C. elegans*.

We found autofluorescent granules to be common in all nematode species we analyzed. Other than *C. elegans*, we examined *O. tipulae* (another *Rhabditis*), *P. pacificus* (an evolutionarily distant Clade V [suborder Rhabditina] species), and 3 Clade IV (suborder Tylenchina) species, namely *S. hermaphroditum*, *P. redivivus*, and *Panagrolaimus* sp. PS1159. In our recent estimation, the divergent time of the Rhabditina and Tylenchina species was estimated to be over 200 million years (Schwarz et al. 2025). We showed that many granules of *O. tipulae* and *P. redivivus* were highly birefringent under Nomarski DIC optics, which rely on circularly polarized light (Fig. 5). In both *O. tipulae* and *P. redivivus*, the appearance of the birefringent granules closely matched the sketches of Cobb (1914). In *O. tipulae*, the distribution of the birefringent granules depends on the stage of the animal and, at least in later stages, has a clear posterior bias. Maupas (1900) reported a similar posterior bias in several of the nematode species he analyzed, including a species now belonging to the *Oscheius* genus (*O. dolichura*, originally *Rhabditis dolichura*). The presence of autofluorescent granules across species and the birefringent granules in *P. redivivus* suggests that “rhabditin granules” are likely to be widespread among nematodes beyond “*Rhabditis*” species.

Analyzing the selected worms, we found that in addition to *C. elegans*, the green flash phenomenon can be observed in at least some stages of *O. tipulae*, *P. pacificus*, *P. redivivus*, and *S. hermaphroditum* (Table 1). In particular, in all 5 species, the phenomenon can be observed during the L4/J4 stage despite the significant difference in the size and shape of these animals, suggesting that the susceptibility of these granules is likely due to their stage-specific biochemical properties and not to factors such as the thickness of the sample (worm). As in *C. elegans*, we found that the susceptibility of the granules differs within each animal, and at least in some species, we found this to be associated with their overall location within the intestine and with other characteristics of the granules, such as the lack of easily observable birefringence. Together, these findings suggest different subpopulations of gut granules in the intestine of the nematodes. This is also the conclusion from another recent independent study of autofluorescence of these granules in *C. elegans* (Hulsey-Vincent et al. 2024).

Further understanding of gut granule green flash may require the characterization of the green and red fluorophores. It is unclear how to best identify these fluorophores, although we would predict that the green fluorophores emit stronger fluorescence under high pH conditions, while the red fluorophores are likely pH-neutral. Whether or not the phenomenon is associated with the presence of the corresponding fluorophores is also uncertain. Regardless, 1 plausible approach to the biochemical causes of the phenomenon would be to compare the molecular content of wild-type and *glo-1(lf)* mutants that lack gut granules, potentially with mass spectrometry, or with fluorescence spectroscopy. Since our results identify the stages in which the green flash can be induced across different species, stage and species-specific samples that contain or lack gut granules could be used to narrow down the candidates. In the non-*C. elegans* species that we analyzed, CRISPR–Cas9 genome modification methods are technically available for *O. tipulae* (Dockendorff et al. 2022), *P. pacificus* (Witte et al. 2015), *S. hermaphroditum* (Cao 2023; Schwartz et al. 2024), and *Panagrolaimus* sp. PS1159 (Hellekes et al. 2023), so knocking out the *glo-1* orthologs may be an effective way to eliminate gut granules in those species. Similarly, one could use proteomics, as we recently reported: we identified gut granule proteins by comparing the proteome of the *C. elegans* wild-type intestine to that of the *glo-1* mutants (Tan et al. 2024).

Overall, we described a visually spectacular fluorescence dynamic phenomenon that involves a type of lysosome-related organelle known as gut granules, which we showed to be common in nematode intestines. We also found that gut granules are likely a collection of distinct subgroups that change during development, and the dynamic fluorescence phenomenon we describe might provide a handle on their eventual characterization.

## Data Availability

Strains are available upon request. Supplemental files available at FigShare (https://doi.org/10.25387/g3.29897192).
